# Experiences of Community Members Engaged in eCPR (Emotional Connecting, Empowering, Revitalizing) Training: Qualitative Focus Group Study

**DOI:** 10.2196/32219

**Published:** 2022-06-30

**Authors:** Amanda L Myers, Mbita Mbao, Arya Kadakia, Shira Collings, Karen L Fortuna

**Affiliations:** 1 Heller School Brandeis University Waltham, MA United States; 2 School of Social Work Simmons University Boston, MA United States; 3 Dartmouth College Hanover, NH United States; 4 National Empowerment Center Lawrence, MA United States; 5 Department of Psychiatry Geisel School of Medicine Dartmouth College Hanover, NH United States

**Keywords:** mental health, trauma, peer support, community mental health education

## Abstract

**Background:**

The United Nations has called for wide-scale community mental health psychoeducation; however, few programs currently exist. Emotional Connecting, Empowering, Revitalizing (eCPR) is a community education and training program developed by individuals with a lived experience of mental health challenges or trauma. It is designed to provide community members with skills and confidence to support someone experiencing mental health challenges.

**Objective:**

This qualitative study aimed to examine the user experiences of diverse community members engaged in eCPR training. This study reviewed their attitudes toward training and opportunities for improvement in future implementations of training.

**Methods:**

eCPR training participants (N=31) were invited to participate in virtual focus groups between June 2020 and July 2020. Data were analyzed using the rigorous and accelerated data reduction method, which converts raw textual data into concise data tables to develop a codebook, and thematic analysis was performed to identify common themes.

**Results:**

The themes identified when analyzing the data included emotional holding and containment, training feedback, principles and practices of eCPR, implementation, connection in a digital environment, skills practice, and shared experiences.

**Conclusions:**

eCPR may benefit individuals from multiple, diverse demographics. It can enhance their ability to connect with others to understand what it means to be with someone who is experiencing a mental health challenge or crisis, to accept their own emotions, and to be confident in being their most authentic self in both their work and personal lives. eCPR may answer the call of the United Nations by bringing opportunities for authenticity and healing to community settings. Exploring the effects of delivering eCPR in communities on individuals experiencing distress is an important next step. This study found that eCPR may be beneficial to many groups of trainees with varying backgrounds and experiences. These findings are important, as they speak to the potential for eCPR to be implemented in a variety of community settings with the intention of working to improve mental health in everyday settings.

## Introduction

### Background

According to the US Department of Health and Human Services’ Substance Abuse and Mental Health Services Administration, mental health disorders are “any mental, emotional, or behavioral disorder that can vary in both severity of impact and impairment” [1]. On the basis of their 2019 National Survey on Drug Use and Health, 1 in 5 (20.6%) adults in the United States had a diagnosed mental health disorder [1]. In 2018, nearly 25% of adults aged ≥18 years with mental health challenges reported an unmet need in receiving mental health treatment [2]. More recently, a nationwide survey in the United States estimated that the rates of substance use have risen 13.3% since the onset of the COVID-19 pandemic, anxiety and depressive disorders have increased by 30.9%, and individuals reported increased trauma and stressor-related disorders related to the pandemic by 26.3% [3]. These challenges faced by individuals during a time of social distancing and lockdown measures highlight the need to increase access to and the delivery of mental health support in the community. Delivering services outside of a clinical setting is essential for task shifting purposes that promote individual recovery without the reliance on an understaffed professional clinical task force.

In light of the increase in mental health challenges and trauma owing to the COVID-19 pandemic, coupled with a shortage of practicing mental health professionals, the United Nations released a report “COVID-19 and the Need for Action on Mental Health” in May 2020 [4]. This report called for wide-scale community mental health psychoeducation in which mailpersons, neighbors, Meals on Wheels workers, and other community members can support one another through a pandemic [4]. This global call to action calls for support outside the clinical environment. However, only a few programs of this type exist. The National Empowerment Center, a nonprofit organization led by Oryx Cohen, MPA, and Daniel Fisher, MD, PhD, has developed a web-based training program in community-based mental health interventions that can be practiced among community members in all settings: Emotional Connecting, Empowering, Revitalizing (eCPR).

Promising evidence indicates that eCPR, a community mental health education and training program, provides community members with the skills and confidence to be with someone who is experiencing mental health challenges and/or a mental health crisis [5]. eCPR is a training program developed by individuals with a lived experience of mental health challenges and/or trauma through the National Empowerment Center and has been described in detail in recent literature [5]. eCPR is based on the recovery model of mental health that uses principles of recovery in its approach toward mental health challenges and trauma [6]. Of note, principles suggest that recovery is person driven, is supported through relationships and social networks (such as peers and allies), and emerges from respect and hope. Moreover, the World Health Organization defines recovery from mental health challenges as “gaining and retaining hope, understanding of one’s abilities and disabilities, engagement in an active life, personal autonomy, social identity, meaning and purpose in life, and a positive sense of self” [7]. eCPR training uses all these principles outside of clinical environments, specifically, to help individuals in community settings gain insight into mental health challenges and how to provide support to others in their community. A recent study has examined the feasibility and effectiveness of virtually delivered eCPR training. Pre- and posttraining surveys were administered to 560 training participants. A total of 151 participants completed both surveys. Participants demonstrated statistically significant improvements in the ability to identify emotions, communicate nonverbally, share emotions, take care of oneself, feelings of belongingness and connection with others, perceived capacity to support individuals, and symptoms and emotions [5].

### Objectives

The purpose of this study is to examine the impact of eCPR on training participants, learn more about their experiences with the training, and evaluate feedback to tailor the implementation of future training. It aims to gain a deeper perspective on the user experience to investigate the underlying mechanisms of the program as well as the priorities and values of various trainees.

## Methods

### Overview

This study used a peer-academic partnership [8]. Peer-academic partnership is a community-engaged approach that aligns with the 11 principles of community engagement that holds both peers and nonpeer academic researchers as experts, which can contribute to the research process [9]. In this instance, peers who are persons who identify as having a lived experience of a mental health challenge and/or trauma contribute to the research process. This partnership has led to a series of studies [5,10-12]. The peer-academic partnership was used in both the development and implementation of this study by developing the research questions, recruitment, retention, and development of the interview guide (Multimedia Appendix 1); conducting focus groups; and interpreting study findings. Preliminary evidence indicates that using such community engagement methods produces more relevant research, increases engagement, expands the uptake of technologies, and improves clinical outcomes compared with traditional research [13].

### Description of eCPR Training

eCPR is designed to be delivered by individuals with lived experiences of mental health challenges including, but not limited to, anxiety, depression, bipolar disorder, schizophrenia, and trauma (ie, peer support specialists and community members) [5] as well as by other community members, such as educators, administrators, and first responders. The 12-hour, 3-day web-based eCPR training included the following modules: (1) connecting with others, (2) using nonverbal communication, (3) cultural empathy across worldviews, (4) learning a trauma-informed approach, (5) addressing feelings of mental distress and thoughts of suicide, (6) empowerment, and (7) revitalization [5]. The aforementioned modules were delivered to the training groups through a variety of teaching methods including role-play, didactic and conversational instruction, and group experiential learning. eCPR trainers participated in 60 hours of education and training with a postexamination to determine eligibility in delivering the eCPR training [5].

### Procedures

#### Recruitment

Trainers emailed individuals who had participated in the eCPR training to inform them about this opportunity. Participants were recruited using a convenience sample of individuals who expressed interest in participating until the desired sample size was reached. The desired sample size was reached at the point where saturation was achieved, and no additional information was presented [14].

#### Informed Consent

Before engaging in the focus groups, the participants were provided with an informed consent form via email to sign electronically. The principal investigator (PI) also read the consent statement aloud at the beginning of each focus group. Individuals were given the opportunity to meet with the PI and ask questions pertaining to the study and the informed consent form. Participants were encouraged to contact the PI at any time with further questions and were notified that they could withdraw their participation at any moment.

#### Demographics

A web-based survey was administered to all participants before eCPR training. Demographic data collected through these surveys were extracted and analyzed for the participants of the focus groups.

### Interview Guide

A total of 2 eCPR focus groups occurred virtually via a Health Insurance Portability and Accountability Act–compliant videoconferencing platform between June 2020 and July 2020 and included 31 participants. The authors conducted semistructured focus groups using an interview guide (Multimedia Appendix 1) developed through an iterative design process. Sample questions included, “What are some of the most valuable things you gained from eCPR training?” “How would you compare your eCPR training to other similar trainings you have had in the past?” and “Do you feel eCPR has changed the way you view someone in emotional distress?” Individuals took part in 1-hour focus group sessions to examine the impact of eCPR training on themselves. No incentives were provided for participation in the study.

### Ethics Approval

The Institutional Review Board of Dartmouth College approved this study (20201271) and was aligned with the 1968 Declaration of Helsinki rules on research ethics [15].

### Statistical Analyses

Descriptive statistics were conducted using SPSS software (version 28.0.0.0; IBM Corp) to describe the demographic characteristics of focus group participants [16]. Focus group data were transcribed and analyzed in Microsoft Excel using the rigorous and accelerated data reduction method for qualitative data coding [17]. This method was chosen for its rigorous team-based approach to organizing qualitative data and streamline analyses. The method converts raw textual data from a general word processing software (Excel) into a more manageable and user-friendly format by producing concise data tables that can be easily reviewed by the team [17]. It was used to generate underlying themes using codes extracted from the qualitative text. The first and second authors performed thematic analysis to identify commonalities in the data-derived codes, which were then translated into the main themes [18]. The authors did not develop a priori codes or hypotheses before coding; instead, codes and themes were developed naturally as they emerged in the review of the data. Codes were assigned to main themes using the *best fit* approach when one or more themes may have been applicable. Furthermore, qualitative data were reviewed by the authors (ALM and MM) to ensure that the results were interpreted for their intended meanings. All results were member checked to maintain the participants’ voices and to ensure that the authors held true to the participants’ viewpoints. Member checking is the process of bringing the authors’ interpretations of the qualitative data back to stakeholders in the population to validate the proper interpretation of the data and resolve any discrepancies that may have emerged [19]. In this case, a qualitative codebook and derived themes were provided to key stakeholders via email. The topics were refined through an iterative process to ensure coherence and adequate data to support each theme.

## Results

### Participants

The 2 focus groups comprised 31 participants each. Participants were aged ≥25 years and identified themselves as peer support specialists and service users with a lived experience of any mental health condition, recovery coaches, hospital staff leaders, family members, mental health clinicians, nonprofit leaders, and nonprofit workers (Table 1). The inclusion criteria for the focus group participants were individuals (1) who participated in and completed the eCPR training between April 2020 and July 2020; (2) who are aged >18 years; (3) who self-identify as experiencing any mental health condition and/or trauma, family members of individuals with mental health conditions, trauma, or physical health conditions, clinicians, nonprofit leaders and workers, and members of the general community; and (4) who were able to provide consent to participate. Individuals who (1) were aged <18 years, (2) deemed cognitively impaired (defined by inability to complete informed consent independently), or (3) had a designated legal guardian were not eligible to participate in the focus groups.

**Table 1:** Demographic characteristics of focus group participants (N=31).

Characteristics			Participants, n (%)
**Gender**			
	Male	7 (23)	
	Female	24 (77)	
**Age (years)**			
	18-24	0 (0)	
	25-34	3 (10)	
	35-44	3 (10)	
	45-54	6 (19)	
	55-64	14 (45)	
	≥65	5 (16)	
**Race and ethnicity**			
	White	23 (74)	
	Black or African American	4 (13)	
	American Indian or Alaska Native	1 (3)	
	More than one race	1 (3)	
	Hispanic or Latino	2 (7)	
**Role^a^**			
	Service user, consumer, or survivor	9 (29)	
	Peer support specialist	8 (26)	
	Recovery coach	3 (10)	
	Family of a person with mental health or substance use issues	8 (26)	
	Clinician	4 (13)	
	Administrator	4 (13)	
	Community member	9 (29)	
	Work for nonprofit	8 (26)	
	Other health service provider	4 (13)	
	Other	8 (26)	

^a^Participants were able to select one or more roles.

Demographic data were obtained for all participants in the focus group (N=31). Most participants were female (24/31, 77%) and within the age range of 55-64 years (14/31, 45%), followed by 45-54 years (6/31, 19%). Of the 31 participants, 23 (74%) identified as White, 4 (13%) identified as Black, 2 (7%) identified as Hispanic or Latino, 1 (3%) identified as American Indian or Alaska Native, and 1 (3.2%) identified as belonging to more than one race. When asked about their roles, 29% (9/31) of the participants identified themselves as service users, 29% (9/31) identified as community members of individuals with mental health concerns, 26% (8/31) identified as peer support specialists, 26% (8/31) identified as a family member of a loved one with mental health or substance use challenges, and 26% (8/31) identified as nonprofit service workers. Other reported roles included recovery coaches, clinicians, administrators, other health service providers, and *other* for those who did not identify with any listed role (Table 1).

We identified 104 codes and 7 themes emerged from the data analysis. The themes identified were emotional holding and containment, training feedback, principles and practices of eCPR, implementation, connection in a digital environment, skills practice, and shared experiences.

### Theme 1: Emotional Holding and Containment

Emotional holding and containment was the most prominent theme, with 25.9% (27/104) of codes related to this theme. Participants reported feeling accepted, heard, and seen. Through emotional holding, the participants reported that they recognized and understood the feelings of the people they were working with. Participants reported feeling held and being able to hold someone. A participant shared the following:

The expression that I resonate with is I felt held, you know, I felt emotionally held and when I am very anxious and fearful scared, being held and seen is super important.

Another participant noted the following:

One of the things I realized that I needed to do more was just being able to be with somebody and not fill the space but let them be and just let them speak and do more listening.

Another participant said the following:

This training was a reminder that it is okay to honor my feelings, that they are telling me something...If I am with someone else, I can feel what I feel and still be connected with that person in their feelings.

The participants discussed the idea of being connected to other people’s feelings and emotions. They reported that they were able to provide a safe container for others and felt emotionally contained during training.

### Theme 2: Training Feedback or Training Format

Participants discussed their experiences with the eCPR training, especially compared with the other trainings they participated in. Training feedback was the second most predominant theme, with 24% (25/104) of codes related to this theme. Feedback from participants about the eCPR training experience varied, with both positive and negative feedback. Participants who liked the unscripted, unconstructed format of the eCPR training reported that the format allowed for them just to be witnesses and to be present in that moment. A participant shared the following comment:

It was a complete kind of like 180 degrees for me from everything else that I have learned as far as the mutuality, as far as the lived, shared, a lived experience and having that peer support...You know, you [are] just there, you [are] just there, however, it goes, you are just with it.

Although several participants preferred the unconstructed and unscripted format, some participants suggested that concrete instructions about eCPR training and planned activities would have helped prepare them. A participant made the following comment:

Since some people like the more nebulous unconstructed freeform way of doing the training maybe for the sake of people like me, give us a little preparation like this is going to be very uncomfortable. You are not going to know what we are talking about. This training is a practice in how to listen. You are going to be listening without doing anything but being emotionally present.

Participants suggested that mindfulness exercises be incorporated at the beginning of the training to prepare them for the emotional holding and containment experienced during training. A participant made the following comment:

The only thing that I would have loved to have seen, and perhaps it could even enhance the training- what I would have liked is that at the beginning of each training session somebody led a guided mindfulness practice.

In addition, the participants suggested that training facilitators should play an active role and direct the training instead of being active participants in the training. A participant noted the following:

For me, it kind of felt a little free flow...I wanted someone responsible for us as a group. Someone to be the concrete space.

The participants also suggested that the PowerPoint used for the training needed to be updated to align further with the content presented in the current eCPR workbook. A suggestion was made to include videos of eCPR in practice or encourage facilitators to role-play and model the skills during the training to provide further examples of practice.

### Theme 3: Principles and Practices of eCPR

In all, 15.4% (16/104) of codes identified related to the principles and practices of eCPR. The participants understood eCPR as a framework for interacting with others. Participants noted that they found training empowering, as it provided freedom to practice with no expectations of fixing or finding a solution to someone’s problems. The training’s lack of concreteness and lack of a script on how to conduct the intervention was described as freeing. Participants found eCPR to be an easy framework that led to positive experiences and deeper connections. A participant shared the following comment:

What this emotional CPR does, it lets you respectfully experience emotions with someone. You let them have their moment and experience it and avoid assumptions and your opinion and what you want...We need this so bad here.

The participants appreciated the lack of need to fix someone or to come up with a diagnosis. Participants noted that the lack of *the need to fix* led to a trusting and safe environment. A participant made the following comment:

eCPR allows us to move beyond the “what is wrong with you” or the stamp of a diagnosis. You say yeah! Of course, you would feel this way; it is hard to feel like this. And going back to that permission to own whatever emotions are coming up and be able to share those.

In comparing the principles and practices of eCPR with those of other trainings the participants had previously taken, participants appreciated that they did not have to memorize a script or any other scripted skills. Rather than learning specific processes and technical content, the participants realized that they just needed to be present and listen deeply to promote recovery through eCPR. A participant shared the following:

Some of the things that we have learned are that you do not have to have the special words...I came away with a different level of confidence because when I was doing the motivational interviewing, I thought maybe I should get some cards or something so that I can do it...but now I do not have to memorize anything to do that

eCPR

### Theme 4: Implementation

In all, 12.5% (13/104) of codes related to implementation were identified. The participants were excited about the training and raised issues related to the feasibility of eCPR implementation in their organization. The participants discussed the importance of obtaining stakeholder buy-in from management, mainly because of the paucity of research on the effectiveness of eCPR on specific populations. Participants noted that there was a need for organizational and personal investments. Organizational investment is needed because the training is costly, and training cannot be conducted in a short period. Staff should be removed from the clock to attend training. A participant made the following comment:

The thing that is challenging is that this is not information that you slap on a PowerPoint and show 30 at a time in 24-hour increments and then call it done...It takes time and to a certain extent involves internal work and kind of a level of reflection for everybody that’s involved.

The same participant also noted that staff participating in the training also had to be ready and willing to share some of their personal issues. This is because the training is structured with the use of real-life problems by the participants, which encourages people to share private information that they usually would not want to share with strangers*.* A participant made the following comment*:*

...and so when you sign up for this training program, you are signing up at a very personal level than anything else you have ever done.

Participants were concerned that eCPR may not be appropriate for some of the clients they work with because individuals with certain disorders lack the ability to connect emotionally based on the nature of the disorder. A participant made the following comment:

I think a concern was it does not fit with all diagnoses. So, it will not necessarily work for everyone, especially when you get into your personality disorders like narcissistic personalities.

The participants also raised concerns about workplace culture and how eCPR could shift how their organizations currently function. A participant commented as follows:

I think it would be a massive culture shift for our hospitals and how to do it [implement eCPR]...and how to get it rolled out that was one of the concerns is it is such a huge culture change.

Implementation concerns related to personnel have also been raised. Some participant organizations were under the impression that they needed to have a peer support specialist on staff to implement eCPR. Participants suggested that agencies would need clear guidance related to a process for implementing eCPR, as most agencies had not yet developed a plan for how eCPR would be incorporated into their organization.

### Theme 5: Connection in a Digital Environment

In all, 10.6% (11/104) of codes related to connecting in a virtual environment emerged. The participants were surprised by the connections they made despite training in a virtual environment. The participants reported feeling safe and trusting enough to open up to others. A participant made the following comment that was echoed by others:

The depth of communication and the sense of trust, even though we were on the computer and had never met these people before...There was a skill there on the human side that was very well done. I felt renewed, refreshed, comforted, safe. I felt very positive.

The participants came into the training skeptical about attending virtual training but were surprised by the more profound sense of connection they had in training with not only people they were familiar with but also people they had never met. A participant commented as follows:

I was really impressed about how successful it could be done by Zoom. I was really coming in pretty skeptical, and when we did the real play where...everybody signed off on the video. It was just the two participants. You know, it really felt like you had that intimacy.

### Theme 6: Skills Practice

Experiential and scenario-based training, especially the *real plays*, was found to be effective in teaching eCPR. Real plays refer to role-playing exercises based on real-life situations that were part of the training. Of the 104 codes, 9 (8.6%) were identified related to skills practice and experiential learning. The participants found it easy to translate eCPR skills into practice compared with the other trainings they had attended. The participants reported using the skills they had learned during training with clients, peers, and friends. A participant commented on their first experience using their eCPR skills outside of the training environment:

I had situations literally within a week of our last session where a client came in, a husband and a wife who was just distraught...I just got certified. I went right into the eCPR mode, so I had a few incidents like that since then...

Another participant shared the following*:*

I had an opportunity to use the eCPR with a co-worker who was expressing frustration, and actually on my part I was thinking, “I do not have to fix this, I just have to be present.” You know, it was more of, I am empathizing with the position that she was in.

Overall, participants found eCPR skills easy to incorporate into their everyday interaction with their clients, partly because eCPR used real-life client problems versus role-play with made-up scenarios as evidenced by this comment from a participant:

The “real plays” versus the role plays were the number one difference between any training because normally it is always a role play, you are given a character you kind of work through the skill set...It was helpful to actually...talk about something of value and not just a thing that you are kind of going through the motions. I think it really brought it home.

### Theme 7: Shared Experiences

The participants’ shared experiences were identified as an emerging theme. Shared experiences are both those that individuals have in common and an individual confiding their experience with another individual or group. Of the 104 codes, 3 (2.8%) related to shared experiences arose. The participants reported that shared experiences led to a deep sense of connection despite being in different locations across the United States. A participant made the following comment:

Everyone [in the training group] was from across the country. So, it was a huge perspective, you know...Having that different perspective across the country actually was a bonding experience for us because it was a shared experience, even though we were not in the same local area going through the same thing.

Through shared experiences, participants were able to connect to deeper emotional levels. A participant commented as follows:

I found myself wanting to interrupt and offer affirmations. However, I held back when I saw how much it meant to her to be able to express how she was feeling and her experience, and when I held back and the more that I listened, we both connected.

## Discussion

### Principal Findings

This study aimed to examine the participants’ experiences after attending the web-based eCPR training. The analysis of the focus group discussions regarding participants’ attitudes toward and experiences with eCPR training highlighted seven main themes: (1) emotional holding and containment, (2) training feedback, (3) principles and practices of eCPR, (4) implementation, (5) connection in a digital environment, (6) skills practice, and (7) shared experiences.

Many participants appreciated the format of the eCPR training and the opportunity to engage in this process with hands-on experience. They expressed feelings of relief in the freedom to practice eCPR teachings and use the eCPR framework without having to *fix* or always be solution driven. The process of connecting in eCPR involves active listening, being present, and fostering a safe environment [5]. The participants found that when the focus was shifted from a solution-driven approach to being present with another person, they were most likely to be in the moment and be with the other person as they experienced their emotions. They appreciated the value of the idea that not everyone is looking for a solution to their experiences, and instead, what they can do is be an active listener while someone talks through and processes what they are experiencing at the moment.

Evidence indicates that participants primarily had a positive training experience. Participants found eCPR training to be a valuable resource for learning new skills when engaging with an individual who may be in distress or experiencing a mental health crisis. These skills included active listening, integrating shared experiences into practice, the ability to connect with others on an emotional level even on a web-based platform, and how to emotionally hold someone in their time of distress and need. Douglas [20] defines containment as “the ability to receive and understand another’s emotional communication, process it, and communicate understanding and recognition back to the other person.” Both holding and containment are essential for healing and recovery [21]. Emotional holding and containment led to participants feeling a deeper sense of trust as they were trusting strangers with personal stories. Not only did the participants report that they felt a sense of emotional holding and containment when participating in the training, but they could actually practice these techniques in their work. eCPR has shown promising evidence for increasing supportive behaviors toward individuals who experience mental health challenges and trauma [5] and can prove useful in both clinical and nonclinical environments such as hospitals, community centers, nonprofits, workplaces, and the community at large.

Multiple participants expressed concern over how the teachings of eCPR would be implemented within their communities—primarily workplaces. However, the concern was not about the actual practices of eCPR but rather about staffing needs and requesting clarification on whether peer support specialists need to be hired to do this work to be able to present to managerial staff. Although it is helpful to have a peer support specialist on the staff, it is not necessary for implementing eCPR. With regard to implementation, participants reported feeling personally invested in this initiative and generally felt that their workplace culture would be accepting of implementing the lessons they learned.

Future research can expand on the impacts reflected in this study by examining the impact of eCPR training on subsets of the population. In addition, research can examine the impact of eCPR training when implemented in various community settings (ie, schools, police stations, medical offices, and apartment complexes). Future studies can focus specifically on the impact of the training has on participants based on their age, mental health diagnosis, role, and race. In addition, future research can be directed to evaluate the effectiveness and impact of in-person eCPR training versus that of eCPR training delivered in a web-based environment. Finally, to answer the call of the United Nations, research could evaluate the impact that implementing eCPR has on community mental health as a whole (ie, levels of anxiety, depression, and distress; feelings of loneliness and isolation; and suicidality). Finally, future studies may attempt to collect more specific details regarding the unique roles and perspectives of the participants. For instance, a clinician who might also be a service user may have differing viewpoints depending on the lens they use. Examining these topics related to eCPR will provide evidence-based insights to inform the future implementation of eCPR training.

### Limitations

This study has some limitations. First, this is the first study to evaluate participants’ experiences in eCPR training. Although the sample size was sufficient to reach saturation, it was not sufficient to stratify the experiential data by demographic characteristics (role, race, and age). In addition, consistent with the peer-academic partnership [8], participants were not asked to disclose any mental health diagnoses; thus, we were unable to stratify the results on that factor either. Focus group participants were recruited as part of a convenience sample, which may have led to a bias in the results. In addition, the training and focus groups were conducted on the web; thus, the results may only be generalizable to web-based environments.

### Conclusions

Although a previous study by the authors evaluated the feasibility and preliminary effectiveness of eCPR training, to the authors’ knowledge, this is the first study to examine participants’ experiences in attending the eCPR training—a community mental health education and training program developed by individuals with a history of mental health challenges and/or trauma, designed to be delivered by such individuals and other community members. In light of the COVID-19 pandemic, the increased or exacerbated mental health challenges individuals face owing to the pandemic and the increased amount of trauma due to COVID-19 fatalities, the United Nations recognizes an increased need for community-based support and wide-scale community mental health psychoeducation. The eCPR training program answers this call by providing a framework for engaging in community-based support without a clinical degree or formal environment. It has been proven to be feasible and effective in its delivery [5]. It is a practice that community members can use to help one another through difficult times and may enhance social connection in a time of disconnection and physical distancing.

## Appendix

Multimedia Appendix 1 Focus group interview guide.

## Abbreviations

eCPR: Emotional Connecting, Empowering, Revitalizing
PI: principal investigator
