# Supplementary material for: Experiences of Community Members Engaged in eCPR (Emotional Connecting, Empowering, Revitalizing) Training: Qualitative Focus Group Study
Source: JMIR Form Res. 2022 Jun 30;6(6):e32219. doi: 10.2196/32219 (PMC9284356; doi:10.2196/32219)
Supplement: Multimedia Appendix 1 [file formative_v6i6e32219_app1.pdf]

Appendix 1. Focus Group Interview Guide

1. What are some of the most valuable things you gained from the eCPR training?

2. How would you compare your eCPR training to other similar trainings you've had in the past?

3. If you do work (including volunteer work, part-time, etc.), have things you gained from the eCPR training been relevant to your work? In what ways?

4. How have you been able to use the eCPR ways of being (skills) in your personal life?

5. Did the training change the way you view yourself and/or your emotions? If yes, how so? Do you feel the training changed you in any way?

6.Do you feel eCPR has changed the way you view someone in emotional distress? In what ways?

7. Have you experienced any negative impact from the eCPR training?

8. How do you see yourself using what you learned from the eCPR training in the future? Are there ways this training could have been more worthwhile?

9. Is there anything else about the eCPR training you would like us to know that we may not have talked about already? Is there anything else you would like to say about your experience with eCPR since the training? What would you most like us to know of your experience with eCPR?
